# Mahmoud Keilani, MB Ch B FRCA

**Published:** 2009

**Authors:** Mohamed Takrouri

**Affiliations:** *Consultant, King Fahad Medical City, Riyadh, Saudi Arabia*

1939-2009

It is with deep sadness that I heard the news of Dr. Mahmoud Keilani’ passing. He was one of the leading anesthetists in Jordan and a great promoter for the cause of Arab anesthetists and for cooperation between Arab countries. He was one of the founders of the Arab Federation of Anesthetists. He served as its Secretary for many years, and then became the Vice President. He was Head of the Jordanian Council for Anesthesia & ICU and the President of the Jordanian Society of Anesthesia several times. His death is a great loss to the profession. He will be well remembered as one of the five who conducted the task of forming the Jordanian Board of Anesthesia. Moreover, he started his Jordan Journal of Anesthesia.

We convey our deep condolences to his family and friends. May Allah bless him and take him in His vast mercy.

